# Formation and Physicochemical Properties of Freeze-Dried Amyloid-Like Fibrils From Pinto Bean Protein

**DOI:** 10.1155/2024/5571705

**Published:** 2024-10-23

**Authors:** Ameneh Allameh, Mohammad Fazel, Nafisehsadat Sheikhan, Mohammad Goli

**Affiliations:** ^1^Department of Food Science and Technology, Najafabad Branch, Islamic Azad University, Najafabad, Iran; ^2^Department of Chemistry, Najafabad Branch, Islamic Azad University, Najafabad, Iran; ^3^Department of Food Science and Technology, Laser and Biophotonics in Biotechnologies Research Center, Isfahan (Khorasgan) Branch, Islamic Azad University, Isfahan, Iran

## Abstract

Amyloid nanofibrils are long and thin strands with cross *β* structures associated by hydrogen bonds. These structures can be formed under suitable conditions commonly at low pH and high temperatures. Fibrillated pinto bean protein isolate (FPBPI) was made by heating pinto bean protein at 85°C in an acidic condition while gently stirring at initial protein solution concentrations of 4 mg/mL, 13 mg/mL, and 21 mg/mL. Freeze-dried FPBPI's physicochemical, structural, and thermal characteristics were assessed, and they were compared with a native pinto bean protein isolate (PBPI) as a control. An increase in Congo red spectral absorption at 544 nm was observed following the fibril formation process. The largest concentration of freeze-dried fibrillated protein exhibited the highest Congo red spectral absorption. Fibrillar proteins' Fourier transform infrared (FTIR) spectrograms with lower wave numbers were seen than the native protein. For native PBPI, transmission electron microscopy (TEM) images were globular in shape, but they changed to long and curly morphologies in fibrillated proteins. FPBPI has a lower melting enthalpy than native protein when measured by differential scanning calorimetry (DSC). With the rising initial protein content, the enthalpy rose. Concurrently, semicrystalline structure for native and fibrillated pinto bean proteins was revealed by X-ray diffraction (XRD) findings. As the original protein concentration grew, so did the crystallinity intensity. Water-holding capacity (WHC) and oil-holding capacity (OHC) of freeze-dried FPBPI were higher than those of native protein. So, fibrillation of pinto bean protein helped it to serve as a good thickener in food industries.

## 1. Introduction

Proteins and peptides that are soluble can be transformed into mature fibers with highly organized structures, including protofibrils. Protein fibrils that resemble amyloid are known as such insoluble fibrillar aggregates [1]. Peptides or proteins can be made into amyloid fibrils in vitro by being subjected to conditions that render them unstable. Many different food proteins can be used to make them [2]. Similar-looking protein nanofibrils can be up to several micrometers long and have a diameter of 5–10 nm. They are linked together by a dense network of hydrogen bonds and contain a high concentration of ß-sheet secondary structure that packs in a cross-ß structure with the *β*-strands parallel to the fibril axis [3]. Both intrinsic (amino acid composition, hydrophobicity, and so on) and extrinsic (temperature, time, pH, ionic strength, shear flow, and so on) factors impact the level and morphology of specific food protein aggregates [1].

Originally used in medical, amyloid and nanofibrils have a long history but have recently been used in food science and nanotechnology [4]. As a result, the term “amyloid” is closely linked to illnesses, and the synthesis of amyloid from various proteins has been associated to a variety of different disorders. Yet, numerous instances of “functional amyloid” have also been discovered, demonstrating that the amyloid structure itself is not harmful to the organism. Such functional amyloid has been discovered in a wide range of animals, including mammals, insects, fungus, bacteria, and most recently, plants. In addition, it has been reported that a variety of proteins from plants, industrial waste streams, or byproducts of food production can form amyloid-like structures in vitro. Protein fibrils that develop in vivo are typically classified as amyloids. The substance is often referred to as amyloid-like if the same proteins are forced to form fibrils in a laboratory setting. These types of fibrils have not yet been associated to any hazardous side effects, which makes it possible to produce protein nanofibrils on a large scale from agricultural or industrial waste streams [5].

With their ability to alter and enhance the physical, structural, and functional properties of food, these food fibrils are regarded as a potential new ingredient in food formulations [2]. Protein nanofibrils made from plant proteins are excellent candidates for use in the creation of new textured foods or plant-based biomaterials due to their mechanical and rheological properties, absence of known toxic effects, and recent emphasis on switching from eating animal-based proteins to plant-based proteins [6]. Functional properties are the desired qualities that a dietary protein should ideally have. They include solubility, water- and oil-holding capacities, emulsifying activity and stability, foaming capacity and stability, gelation, and texturizability. These functional qualities, which have an impact on the final product's attributes, are what determine how well protein products are utilized [7].

To the best of our knowledge, among legume proteins, pinto bean protein fibrillation has not been the subject of published data, and studies pertaining to this topic on other protein sources have focused on fibrillated protein suspension [4, 7–12]. Using varying initial protein solution concentrations in freeze-dried form, we confirmed the fibrillation of the pinto bean protein supply in this study and assessed it using Congo red spectrum analysis. The morphology of freeze-dried fibrils was identified using transmission electron microscopy (TEM), and changes in secondary structure were assessed using Fourier transform infrared (FTIR). Using differential scanning calorimetry (DSC), thermal characteristics were examined. X-ray diffraction (XRD) was used to analyze the crystallinities of native and fibrillated proteins that had been freeze-dried. Finally, functional properties of the freeze-dried fibrillated pinto bean protein were assessed for use as a food ingredient.

## 2. Materials and Methods

Arak, an Iranian seed-improving company, provided the pinto bean (*Phaseolus vulgaris*; C.V. Saleh) with a mean protein content of 20.8/100 g. We bought Congo red, sodium hydroxide, and hydrochloric acid from commercial sources (Merck, Germany).

A bean grain grinder was used to make pinto bean flour, which was then sieved through a 0.5 mm sieve.

### 2.1. Preparation of pinto bean protein isolate (PBPI)

Munialo et al.'s method was used to manufacture the PBPI. In brief, pinto bean flour dispersions in water (10% w/v) were adjusted to pH 8 with 1M NaOH and incubated at room temperature. The sample was then centrifuged at 10000 g for 20 min. To precipitate the protein, the supernatants were mixed and the pH was adjusted to 4.8 using 1M HCl. The mixture was then incubated for 2 h with continuous stirring before being centrifuged at 10000 g for 25 min. The protein was then resuspended in deionized water in a 1:3 ratio after the supernatant was removed, and the pH was then adjusted to 8 using 2M NaOH. To get rid of any remaining insoluble materials, the solution was centrifuged (10000 g, 25 min) after being incubated at room temperature [7]. The dried protein was lyophilized from the supernatant and kept at −20°C until use. The protein contents of three replicates of the lyophilized sample (pinto bean protein) were calculated 87% using the Kjeldal method.

### 2.2. Fibrillar Protein Preparation

In order to produce fibrils, deionized water was mixed with three different quantities of freeze-dried protein (5, 15, and 25 mg/mL) together with sodium azide (0.02%) and was kept for hydration overnight at 4°C. With 1 M HCl, the pH of the supernatants was brought down to 2. The dispersion was then heated for 20 h at 85°C with a gentle stirring speed of 300 rpm on a plate heater stirrer. The fibrillation process was then stopped by rapid cooling in an ice bath [7]. The biuret method was used to determine the final protein concentrations in the dispersions, which were 4, 13, and 21 mg/mL. Before investigations, produced fibrillar dispersions were freeze-dried or chilled at 4°C for the Congo red spectral analysis.

### 2.3. Fibrillar Protein Characterizations

#### 2.3.1. Congo Red Spectral Analysis

According to Tang's approach in 2010, Congo red binding assay was carried out in a UV-Vis spectrophotometer (Photonix AR 2017) in the scanning range (400–700 nm) to validate the existence of pinto bean protein fibrils. Basically, 10 mM of phosphate buffer saline (PBS) (pH 7.4) was used to dissolve the dye in order to create a stock solution of 10 mM Congo red. For baseline correction, PBS was utilized. Prior to spectrum analysis, 100 *μ*L of each fibrillated protein (with concentrations of 4, 13, and 21 mg/mL) or the native protein (with a concentration of 20 mg/mL) were combined with 1 mL of fresh working Congo red stock solution (0.1 mM). As a blank, Congo red working solution was utilized [8].

#### 2.3.2. FTIR

An Avatar FTIR spectrophotometer was used to obtain FTIR spectroscopic data for freeze-dried materials at intervals of 4000-500 cm^−1^. The amide I region's secondary protein and fibril structures have been identified (1700–1600 cm^−1^) [1].

#### 2.3.3. TEM

Protein fibrils were imaged using TEM (model: Philips CM30). A droplet of 1 *μ*g/mL native PBPI or fibrillated pinto bean protein isolate (FPBPI) in pH 2 tris buffer, was dropped on a carbon-coated copper grid. Excess water was removed. Finally, the samples were negatively stained using uranyl acetate and gave time to have been dried prior to observation [13].

#### 2.3.4. DSC

Using Setaram DSC 131 and the Farrokhi et al.'s method, the thermal denaturation of natural and freeze-dried fibrillated pinto bean protein (at three different concentrations) was assessed. Each sample was weighed in a 40 *μ*L aluminum pan at 4–6 mg. The pans were hermetically sealed and heated at a rate of 10°C/min between 20°C and 200°C [14].

#### 2.3.5. XRD

XRD of native and freeze-dried fibrillated materials was performed using an X-ray diffractometer (Model, Philips PW3040). The device with copper tubes was initially set up with an anode current of 30 mA and an accelerating voltage of 40 kV. Cu-K*α*1 radiation with an estimated wavelength of 0.154 nm was applied to all samples. With a step size of 2*θ* = 0.05 and a diffraction angular range of 10°–90° (2*θ*), the samples were analyzed [14].

### 2.4. Native and Fibrillated Freeze-Dried Pinto Bean Protein Functional Properties

#### 2.4.1. Solubility

Zhongh and Xiong's method was used to measure protein solubility [15]. Aqueous dispersions of native or fibrillated freeze-dried protein isolate (5.0 mg/mL) were solubilized, with pH adjusted to 6.5 (pH in which native or fibrillated powders can be used as an ingredient in food products) and centrifuged at 10,000 g for 15 min at room temperature. The biuret assay kit was used to calculate the protein concentration in the supernatant.

#### 2.4.2. Emulsifying Properties

Using an ultrasonic homogenizer, sunflower oil (2 mL) and 6 mL of 0.1% freeze-dried native or fibrillated protein solution (pH = 6.5, stirred overnight) were combined. At 0 and 10 min after homogenization, parts of the emulsions measuring 50 *μ*L each were pipetted from the bottom of the container. 5 mL of a 0.1% SDS solution were used to dilute each serving. These diluted emulsions' absorbances were measured at 500 nm. The emulsifying activity index (EAI) and the emulsion stability index (ESI) were calculated using the absorbances obtained immediately (A0) and 10 min (A10) after emulsion formation as follows:

(1) $$\begin{array}{c} \text{EAI}\left(\frac{m^{2}}{g}\right)=\frac{2T\left(A0\times \text{dilution factor}\right)}{C\times \varphi \times 10000}, \end{array}$$

where *T* = 2.303; A0 = absorbance measured immediately after emulsion formation; dilution factor = 100; *C* = weight of protein/unit volume (g/mL) of aqueous phase before emulsion formation; and Φ = oil volume fraction of the emulsion.

(2) $$\begin{array}{c} \text{ESI}=\frac{A0\times ∆t}{∆A}, \end{array}$$

where ∆*t* = 10 min and ∆*A* = *A*0 − *A*10 [16].

#### 2.4.3. Water Absorption Capacity (WAC)

Farrokhi et al. defined WAC as the amount of water absorbed per gram of protein on a dry basis, expressed as a percentage [14].

#### 2.4.4. Oil Absorption Capacity (OAC)

OAC was calculated as the amount of sunflower oil absorbed per gram of natural or fibrillated protein that was freeze-dried [14].

### 2.5. Statistical Analysis

The experiments were carried out using a completely random design. Using SPSS statistical software, one-way ANOVA was used to analyze the data at a 5% probability level (*p* < 0.05). Each measurement was carried out three times, and LSD was used to determine the mean significant difference.

## 3. Results and Discussion

### 3.1. Congo Red Spectral Analysis

Proteins containing amyloid are stained using Congo red. This relatively unique staining has drawn notice for two different reasons. First, Congo red is frequently employed for practical reasons to show amyloid in pathological specimens. Second, the binding of Congo red to amyloid has been exploited as a heuristic to shed light on this significant protein group's macromolecular structure [17]. A maximum in the absorbance spectrum for Congo red with a diazo sulfonate structure is at 498 nm. Yet, depending on the protein structure and environment, a red or blue shift happens when Congo red binds to peptides or proteins [18].

**Figure 1:**
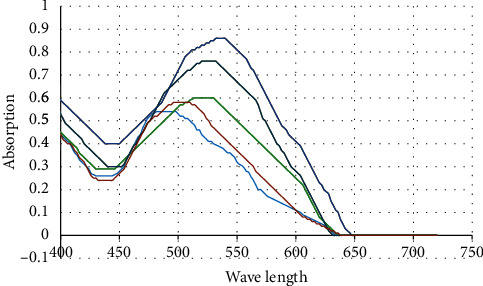
Dark blue curve represents the fibrillated pinto bean protein isolate (FPBPI) with a 21 mg/mL initial protein concentration; dark green curve represents the FPBPI with a 13 mg/mL initial protein concentration; light green curve represents the FPBPI with a 4 mg/mL initial protein concentration; brown curve represents the native pinto bean protein isolate; and light blue curve represents the working standard of Congo red solution.

Figure 1 shows the Congo red absorption spectra of native protein and its nanofibril forms at various starting protein concentrations. The protein content affects the speed and morphology of aggregation by influencing interactions between particles in a solution [19]. Interestingly, the native protein form displayed lower absorptions in the 530–536 nm region than that of the two protein solution concentrations of 13 mg/mL and 21 mg/mL. This suggested that greater *β*-sheet structure contents were present in fibrillated samples. As the original protein content was larger, the Congo red absorption increased. More proteins were, therefore, absorbed into the fibrils and resultant intermolecular *β*-sheets for a protein concentration of 21 mg/mL.

Congo red absorbance spectra in solutions with and without fibrillary protein were compared by Ye et al. They demonstrated significant changes in the Congo red absorption spectra, which suggested fibrillary whey protein isolate had amyloid-like morphologies [20]. Congo red's behavior in a fibrion over time was demonstrated by Ahrami, Khatami, and Heli under fibrillation conditions. They showed that the absorbance intensity rose and the absorbance maxima had a red shift from the end of the fibrillation time following the lag phase of fibril production [18].

### 3.2. FTIR Analysis

**Figure 2:**
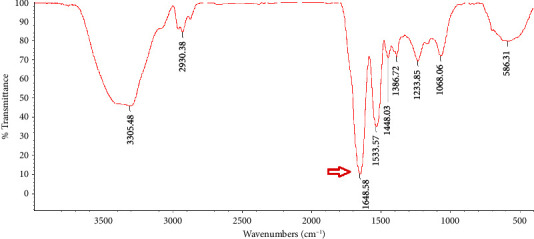
Freeze-dried native pinto bean protein isolate Fourier transform infrared (FTIR) spectra.

**Figure 3:**
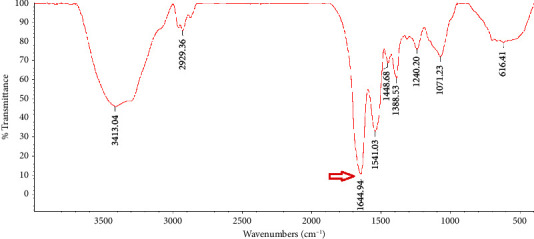
Freeze-dried fibrillated pinto bean protein isolate Fourier transform infrared (FTIR) spectra with 4 mg/ml initial protein concentration.

**Figure 4:**
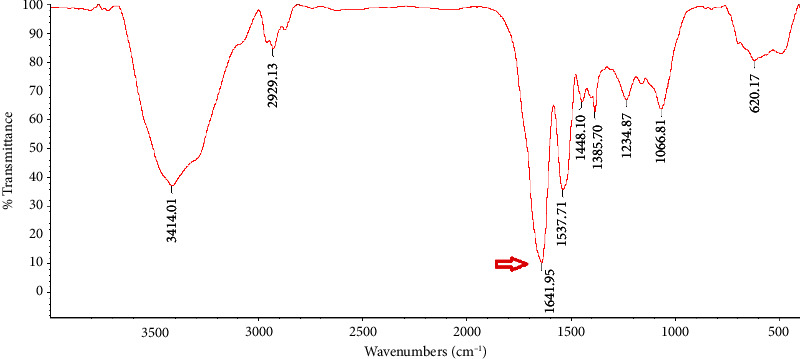
Freeze-dried fibrillated pinto bean protein isolate Fourier Transform Infrared (FTIR) spectra with 13 mg/ml initial protein concentration.

**Figure 5:**
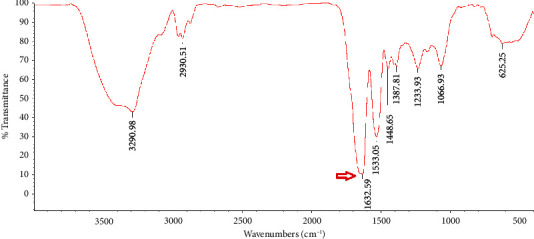
Freeze-dried fibrillated pinto bean protein isolate Fourier transform infrared (FTIR) spectra with 21 mg/ml initial protein concentration.

A valid technique for examining protein secondary structures is FTIR spectroscopy, which primarily works by identifying the amide I, ІІ, and ІІІ regions [14]. In the recent years, IR methods have been reevaluated as a viable tool for tracking the production of amyloid fibrils [21]. Freeze-dried natural PBPI and FPBPI FTIR spectra are shown in Figures 2, 3, 4, and 5.

Wave numbers at 1700–1600 cm^−1^ (related to *c* = *o* stretching of the peptide bond) are assigned to amide I region [22]. The changes in the protein's secondary structure during the process of fibril production are extremely noticeable in the amide I band [23]. With an initial protein content of 21 mg/mL, the FPBPI sample displayed *β*-sheet structure, which was identified by a prominent peak at around 1632 cm^−1^ (Figure 5). As shown in Figure 2, a band at 1648 cm^−1^ in the FTIR analysis of native sample is assigned to random coil [24] shifted to 1644, 1641, and 1632 cm^−1^ in fibrillated sample with initial protein concentrations of 4 mg/mL, 13 mg/mL, and 21 mg/mL, respectively. Ye et al.'s studies revealed a change toward smaller wavenumbers in the amide I region (1580–1700 cm^−1^) in fibrillated whey protein isolate as compared to nonfibrillated one, indicating a greater concentration of ß-sheet structure [20], which validates our findings of main peaks changing to lower wave numbers. This change is more significant at initial fibrillated protein concentrations of 21 mg/ml. Li et al.'s FTIR studies showed shifting to lower frequency peaks after protein fibrillation [4].

According to Farrokhi et al., a band at 1652 cm^−1^ for the control sample assigned to *α*-helix shifted to 1630 cm^−1^ for nanofibrillated samples. This band (1630–1640) is linked to the presence of a intermolecular *β*-sheet structure. Hydrogen bonds that are important for stabilizing the structure of native protein are destroyed during fibrillization, resulting in the creation of an intermolecular *β*-sheet structure [14]. Yet, in Mohammadian and Madadlou's FTIR spectroscopy experiments, fibrillated whey protein moved from 1644 cm^−1^ in the native protein amide I region to 1645 cm^−1^ in the fibrillated form. They proposed that this change to higher wave numbers resulted in a more extensive breakage of hydrogen bonds rather than their creation intermolecularly throughout the fibrillation process [25].

### 3.3. TEM

**Figure 6:**
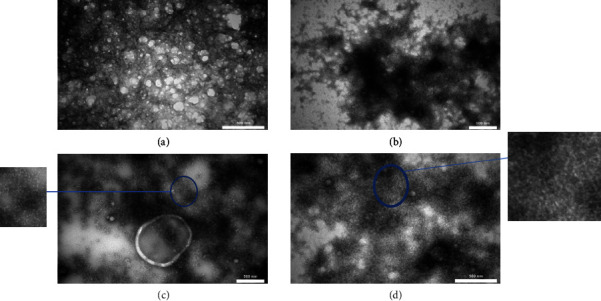
Transmission electron microscopy (TEM) image of (a) freeze-dried native pinto bean protein isolate, (b) freeze-dried fibrillated pinto bean protein isolate fibrillated at 4 mg/mL initial protein concentration, (c) freeze-dried fibrillated pinto bean protein isolate fibrillated at 13 mg/mL initial protein concentration, and (d) freeze-dried fibrillated pinto bean protein isolate fibrillated at 21 mg/mL initial protein concentration.

Figure 6 shows TEM images of freeze-dried native PBPI and FPBPI (the original figures are supplied in supporting information numbered S1–S4 for Figure 6 parts A to D, respectively). Microscopic imaging revealed that little fibril is generated at 4 mg/mL protein concentration. It appears that the critical aggregation concentration (CAC) was not attained, as evidenced by TEM. The CAC for fibril production, according to Kroes-Nijboer, Venema, and Linden, is a significant parameter since it contains information on the thermodynamics and thus the driving mechanism behind fibril creation. The incorporation of peptides into fibrils was discovered to be the rate limiting step for fibrillar development at low protein concentrations [3]. After 20 h of heating, amyloid-like fibrils were produced at protein concentrations of 13 mg/ml and 21 mg/ml. This finding is consistent with Congo red spectral analysis, which revealed a significant increase in absorption at 530–536 nm after fibrillation with greater (13 and 21 mg/mL) initial protein concentrations.

### 3.4. DSC

DSC is an experimental technique used to determine the energetics of biological macromolecule conformational changes [26]. Heat can melt and disintegrate protein aggregates of various proteins, including amyloid fibrils, resulting in distinctive endothermic changes in DSC measurements [27].

**Figure 7:**
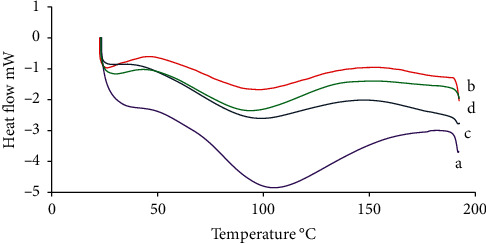
Differential scanning calorimetry (DSC) thermograms of (a) freeze-dried native pinto bean protein isolate, (b) fibrillated pinto bean protein with 4 mg/ml initial protein concentration, (c) fibrillated pinto bean protein with 13 mg/ml initial protein concentration, and (d) fibrillated pinto bean protein with 21 mg/ml initial protein concentration.

Figure 7 shows DSC thermograms of freeze-dried native and fibrillated protein (DSC specifications of graphs A–D in Figure 7 are provided in supporting information numbered S5–S8, respectively). The presence of an endothermic peak in all samples demonstrates the importance of hydrogen bonds and van der Waals interactions in native protein and fibrillated forms [27]. The melting enthalpy of FPBPI was decreased. Reduced ΔH in freeze-dried fibrillated samples compared to native samples could be due to hydrophobic interactions between protein molecules and their effect on ΔH reduction [28]. According to Farrokhi, Milani, and Golimovahhed, the melting enthalpy of native whey protein was higher than that of nanofibrillar structures due to the highly ordered structure [28].

Wang et al. found an endothermic peak and an exothermic peak in the native soy protein isolate but only an exothermic peak in the hydrolyzates [1]. Farrokhi et al. demonstrated that both native globular and nanofibrillated whey proteins were glassy at ambient temperature and exhibited a glass to rubber transition during DSC heating. In their tests, the melting enthalpy of native whey protein was higher than that of nanofibrillar structures, which is consistent with our findings [29].

DSC profiles of fibrillated protein at various doses were found to be closely linked. It can be explained that there exists short-range molecular ordering that DSC would identify as ordered structures due to fibril disintegration during freeze drying and the presence of nonaggregated peptides [29].

### 3.5. XRD

The structure of powders in food production has a significant impact on their stability, functioning, and applicability. Only a few studies have been published on the use of the XRD technology for food characterization [30]. When X-ray light strikes the lattice planes of crystalline materials, it scatters only in certain directions, resulting in high intensity narrow peaks, whereas the random orientation of atoms in amorphous materials causes incident X-ray light to be scattered in random directions, resulting in broad peaks [29]. XRD data can be used to understand crystal polymorphism and the fraction of crystalline and amorphous content [31, 32].

**Figure 8:**
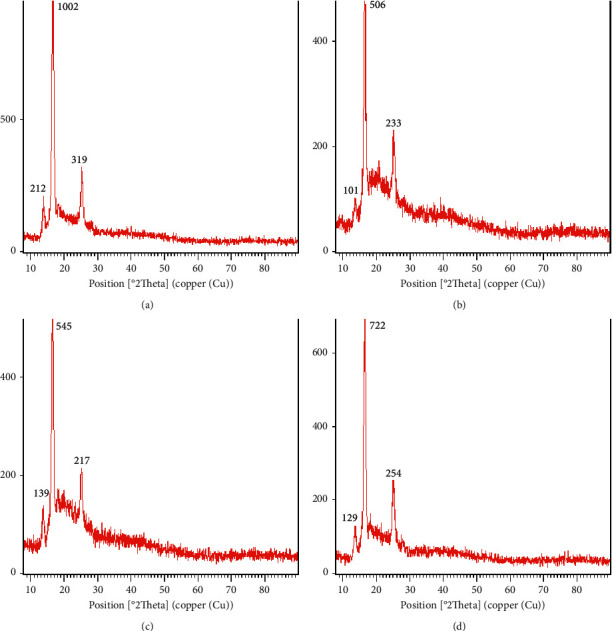
X-ray diffractograms of (a) freeze-dried native pinto bean protein, (b) fibrillated pinto bean protein with 4 mg/ml initial protein concentration, (c) fibrillated pinto bean protein with 13 mg/ml initial protein concentration, and (d) fibrillated pinto bean protein with 21 mg/ml initial protein concentration.

Figure 8 shows that at different concentrations, either the native or the fibrils had semicrystalline structures with strong peaks at 2Ө of 14, 17, and 25, with varying degrees of crystallinity (these figures are also available in supporting information files numbered S9–S12 for Figure 8 parts A–D, respectively). The intensity of native pinto bean protein was highest in the sharp peaks. Their intensities dropped as a result of fibrillation. Yet, raising the initial protein concentration increased the intensities of sharp peaks. When Congo red spectral analysis of fibrillated proteins is combined with XRD diffractograms, enhanced crystallinity intensity is found to increase with increasing Congo red spectral absorption at 530–536 nm. Farrokhi et al. discovered a semicrystalline structure in fibrillated whey protein using XRD, and the extent of the crystalline portion increased with protein concentration [29].

### 3.6. Native and Fibrillated Pinto Bean Protein Functional Properties

#### 3.6.1. Solubility

After centrifugation, the milligrams of protein in either native or fibrillated protein dispersions (5 mg/ml) are shown in Table 1. PBPI solubility at pH = 6.5 reduced after fibrillation for 13 mg/ml and 21 mg/ml initial protein concentrations but was not significantly (*p* < 0.05) changed when 4 mg/mL initial protein concentration used. The solubility of freeze-dried with the initial protein concentration of 21 mg/mL was not statistically different (*p* < 0.05) from the sample with 13 mg/ml initial protein concentration.

According to Mohammadian and Madadlou, fibrillation lowered the solubility of both whey protein isolate suspension and whey protein enzymatic hydrolyzate, particularly at close to isoelectric pH levels [33]. Wang et al., found that heating duration increased soy protein solubility near isoelectric pH (4.8) [1]. Yet, when the pH was far from the isoelectric point, soy protein isolate was more soluble than certain hydrolyzates. Farrokhi et al. found that freeze-dried native whey protein isolate had the highest solubility at all pH levels when compared to fibrillated samples [14]. Fibrillization was previously shown to reduce the solubility of whey protein isolate, which was explained by the development of stranded nanofibrils and an increase in protein surface hydrophobicity [14].

#### 3.6.2. Emulsifying Activity

Protein solubility, pH, droplet size, net charge, interfacial tension, viscosity, and protein structure all influence emulsifying properties [34]. The emulsifying activity of native and fibrillated protein isolates is shown in Table 1. The EAI and ESI of freeze-dried fibrillated protein were much lower than those of native protein, most likely due to heating time making fibrils coarse enough to cover emulsion droplets. However, by increasing initial protein concentration, EAI and ESI were significantly increased. As a result, fibrillated pinto bean protein may be ineffective as an emulsifier in emulsion-based foods.

According to Congo red spectral results, almost no fibril is formed, when the initial protein concentration is 4 mg/ml. EAI and ESI of 4 mg/ml initial protein concentration are lower than those of fibrils generated by 13 and 21 mg/mL initial protein concentrations. It seems that peptides in 4 mg/ml heated protein solution sample are not well organized to decrease the interfacial tension between oil and water phases. So, fibrils formed in higher concentration protein suspensions are more efficient in decreasing surface activity and increasing emulsion stability than the hydrolyzates resulted from 4 mg/mL initial protein concentration suspension.

It was expected that EAI would be lowered due to the decreased solubility of fibrillated protein. Tang et al. demonstrated that protein solubility is directly related to functions such as emulsifying, foaming, viscosity, and sensory characteristics [35]. Serfert et al. discovered that the emulsifying activity of *β*-lactoglobulin fibrils was greater than that of native protein [36]. We employed freeze-dried fibrils, whereas Serfert et al. and other investigators, including those on the emulsifying action of fibrillated proteins, used fresh fibril dispersions to create emulsions [36]. Straight fibrils were likely fragmented into short rods during freeze-drying and rehydration, affecting the emulsifying characteristics [37].

#### 3.6.3. Water-Holding Capacity (WHC)

The ability to resist water releasing from the protein's three-dimensional structure is referred to as the WHC (often also described as water binding or water absorption capacity) [38]. Table 1 displays the WHC of PBPI and fibrillated samples. Water is considerably (*p* < 0.05) more absorbed in fibrillated protein isolates than in native protein isolates. Its absorption capacity was greater in a heated protein suspension with 13 and 21 mg/mL initial protein concentration than that in the heated protein suspension with 4 mg/mL concentration.

The quantity and location of charged, reactive sulfhydryl, hydrophobic, and hydrophilic residues increased in heat-induced unfolded proteins, providing the potential for molecular rearrangements and intermolecular interactions to produce strong water binds. Furthermore, because these molecules are more hydrophilic, the presence of nonaggregated proteins and free peptides plays a key role in boosting WHC [29]. The findings of Farrokhi et al. confirm that the fibrillated whey protein isolate sample has a higher water absorption capacity than the original one [14].

#### 3.6.4. Oil-Holding Capacity (OHC)

Surface area influences oil absorption, indicating a relationship between globular protein hydrophobic interactions and the number of monolayer oil binds. Denaturation and fibril formation convert compact globular protein to long-stranded fibrils, causing previously buried hydrophobic peptide bonds to be exposed. The structure of the heat-induced fibrillar network greatly influences oil binds because oil may effectively bond with the exposed nonpolar structure [14]. Table 1 reveals that the FPBPI held more oil than the native protein. OHC increased considerably by extending the initial protein suspension concentration from 4 to 21 mg/mL. OHC was not significantly (*p* < 0.05) different when the 4 mg/mL initial protein suspension concentration was used. The sample with the highest quantity of OHC was the fibrillated protein heated with 21 mg/mL initial protein suspension concentration. Because of their greater oil absorption ability, nanofibrillar proteins are appropriate for use in food processing, such as bread items. Farrokhi et al.'s research confirmed the superiority of fibrillated whey protein isolate's OHC over the native one [14].

## 4. Conclusions

It can be concluded that pinto bean protein was successfully used to create fibrils at pH 2.0. The original protein concentration of 4 mg/mL of fibrillated pinto bean protein concentration did not produce fibrillar aggregates. However, raising the protein concentration to 13 mg/mL led to the development of fibrils, and the fibrils were successfully generated at the initial protein concentration of 21 mg/mL. The degree of crystallinity and the fibril melting enthalpy increased with an increase in the initial protein content. OHC and WHC of freeze-dried fibrillated pinto bean protein with 13 and 21 mg/mL initial protein suspension concentration were greatly improved than the native protein. So, these products can be used to thicken food made from plants or to stop syneresis or juiciness.

### 4.1. Future Outlines

In the future the following research studies can be done:

• Adding various amounts of salts to study the ionic strength effect on the fibrillation process
• Possibility of new protein sources to fibrillate
• Practical and other functional applications of freeze-dried fibrillated protein from other sources
• Applying freeze-dried protein fibrils in food products and evaluating the product sensory and textural properties
• Investigation of freeze-dried fibrils digestion in human body.

## Figures and Tables

**Table 1:** Functional properties of native and fibrillated pinto bean protein isolate (PBPI).

	Solubility	EAI	ESI	WHC	OHC
Native PBPI	5.0^a^ ± 1	78.4^a^ ± 1.08	79.81^a^ ± 1.57	1.21^c^ ± 0.07	1.79^c^ ± 0.03
Fibrillated pinto bean protein with 4 mg/ml initial protein concentration	5.67^a^ ± 0.57	20.32^d^ ± 0.87	25.45^d^ ± 0.3	1.54^b^ ± 0.06	1.69^c^ ± 0.06
Fibrillated pinto bean protein with 13 mg/ml initial protein concentration	3.67^bc^ ± 0.57	46.19^c^ ± 0.34	46.18^c^ ± 0.33	1.85^a^ ± 0.05	2.23^b^ ± 0.08
Fibrillated pinto bean protein with 21 mg/ml initial protein concentration	3.33^c^ ± 0.57	63.74^b^ ± 1.46	59.45^b^ ± 0.27	1.96^a^ ± 0.11	2.65^a^ ± 0.06

*Note:* Means ± SD (*n* = 3) with different letters in each column indicate significant difference (*p* < 0.05).

## Data Availability

Data used to support the findings of this study are available from the corresponding author upon reasonable request.

## Supporting Information

Supporting information are provided in attachments. Figures S1–S4 of supporting information are original images of TEM. Figures S5–S8 are DSC specifications of Figure 7. Figures S9–S12 of supporting information are png image files of XRD graphs.
