# Supplementary material for: Formation and Physicochemical Properties of Freeze-Dried Amyloid-Like Fibrils From Pinto Bean Protein: Amyloid-Like Fibrils From Pinto Bean Protein
Source: Int J Anal Chem. 2024 Oct 23;2024:5571705. doi: 10.1155/2024/5571705 (PMC11524705; doi:10.1155/2024/5571705)

SETARAM Instrumentation KEP Technologies **Figure :** 1 8/9/2021 **Experiment :** Allameh-Ameneh-N-0518 **Atmosphere :** 1:Air, 2:N2

Procedure : Procedure 8/9/2021 7:57:45 AM **Mass :** 6.31 (mg)

DSC131 Evo (No option) **Zone name :** 1 Standard zone **Molar mass :** - admin

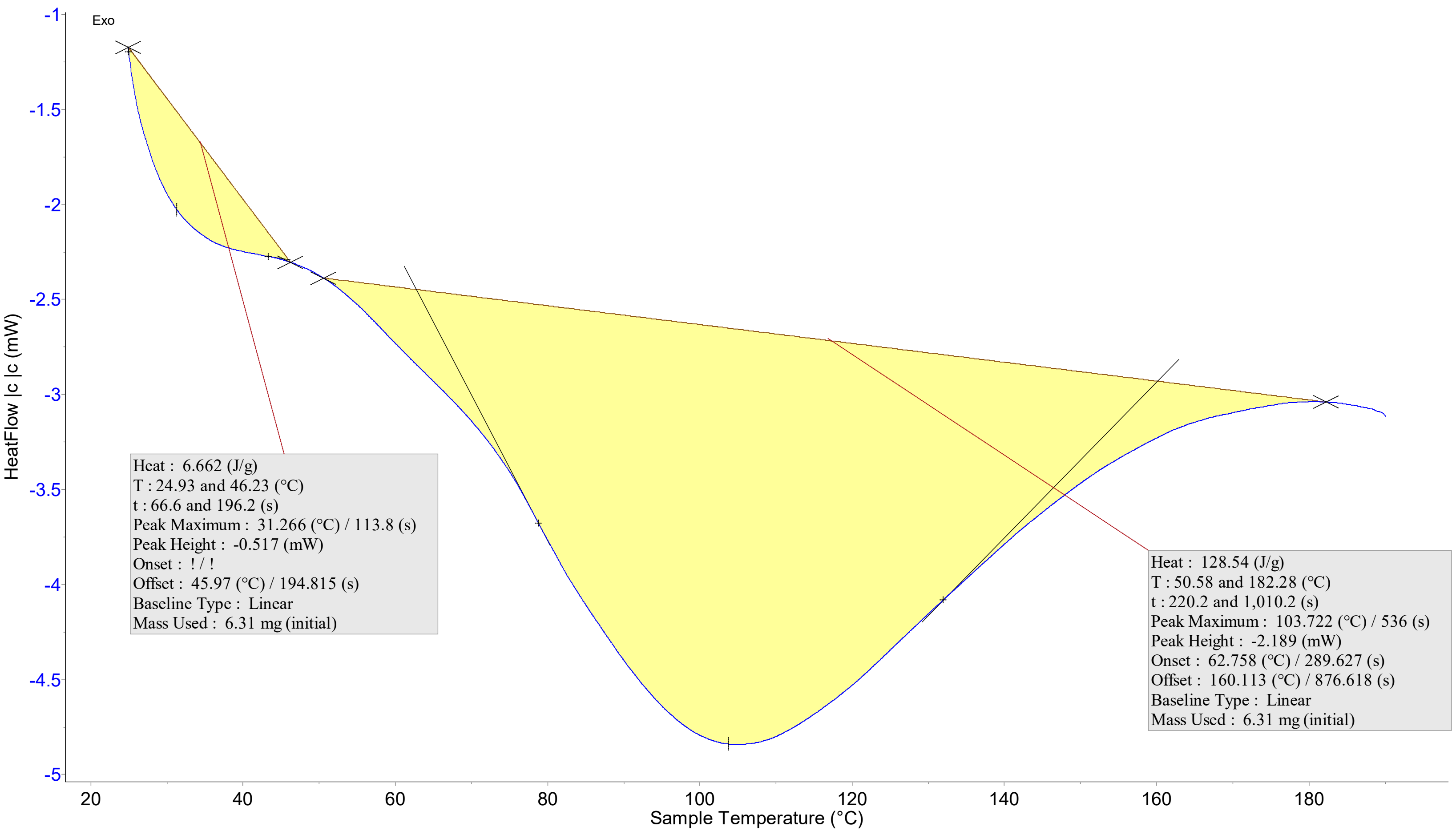

Supplement: Supporting Information — Additional supporting information can be found online in the Supporting Information section. [file 5571705.f1.zip › S5.pdf]
