# Supplementary material for: Formation and Physicochemical Properties of Freeze-Dried Amyloid-Like Fibrils From Pinto Bean Protein: Amyloid-Like Fibrils From Pinto Bean Protein
Source: Int J Anal Chem. 2024 Oct 23;2024:5571705. doi: 10.1155/2024/5571705 (PMC11524705; doi:10.1155/2024/5571705)

SETARAM Instrumentation KEP Technologies	**Figure :** 1	**Experiment :** Allameh-Ameneh-F1-0518	**Atmosphere :** 1:Air, 2:N2
	8/9/2021	**Procedure :** Procedure 8/9/2021 8:07:57 AM	**Mass :** 4.01 (mg)
DSC131 Evo (No option)		**Zone name :** 1 Standard zone	**Molar mass :** - admin

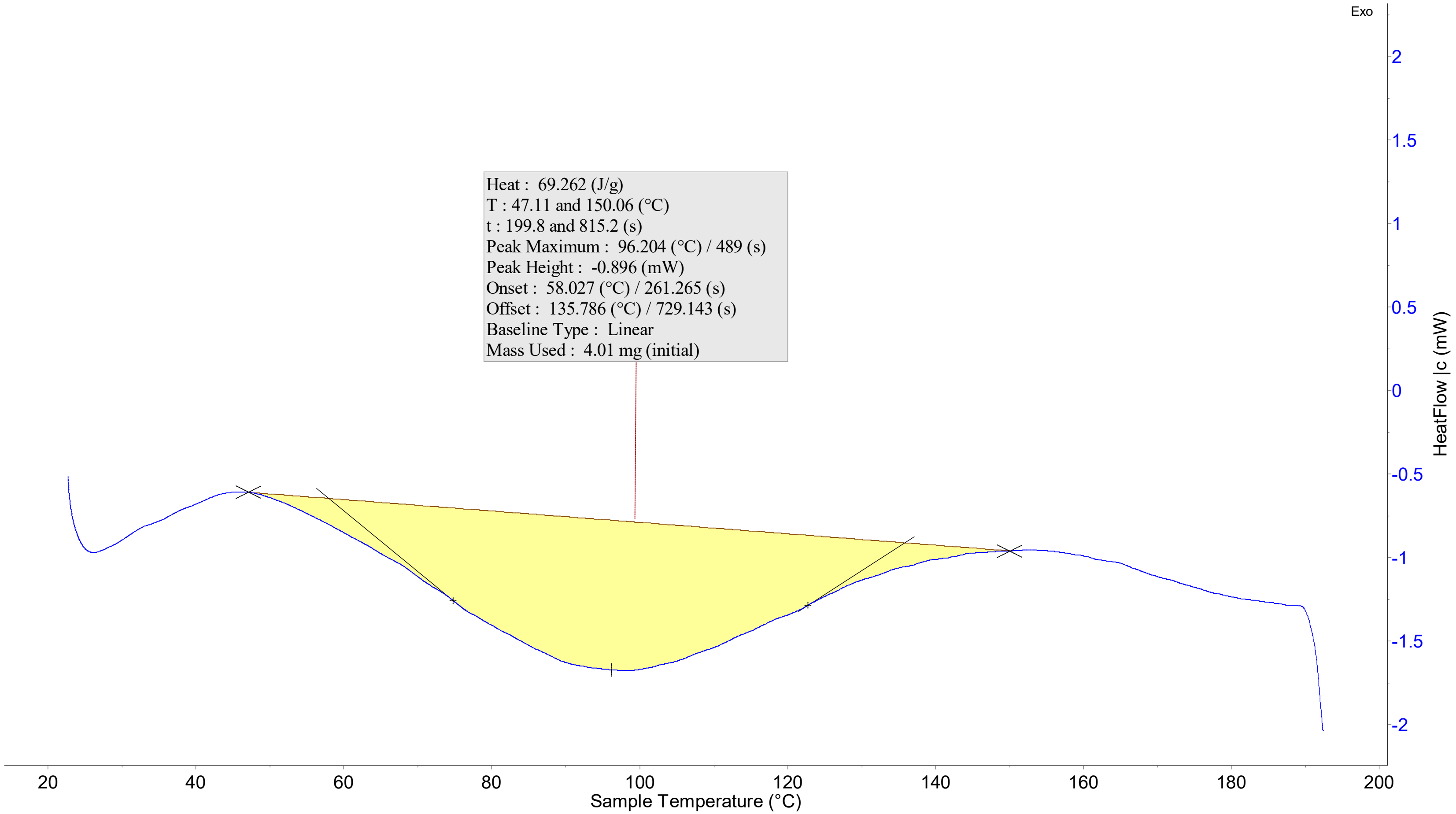

Supplement: Supporting Information — Additional supporting information can be found online in the Supporting Information section. [file 5571705.f1.zip › S6.pdf]
