# Supplementary material for: Formation and Physicochemical Properties of Freeze-Dried Amyloid-Like Fibrils From Pinto Bean Protein: Amyloid-Like Fibrils From Pinto Bean Protein
Source: Int J Anal Chem. 2024 Oct 23;2024:5571705. doi: 10.1155/2024/5571705 (PMC11524705; doi:10.1155/2024/5571705)

SETARAM Instrumentation KEP Technologies

**Figure :** 1
8/9/2021
DSC131 Evo (No option)

**Experiment :** Allameh-Ameneh-G-0518
**Procedure :** Procedure 8/9/2021 9:57:54 AM
**Zone name :** 1 Standard zone

**Atmosphere :** 1:Air, 2:N2
**Mass :** 4.02 (mg)
**Molar mass :** -

admin

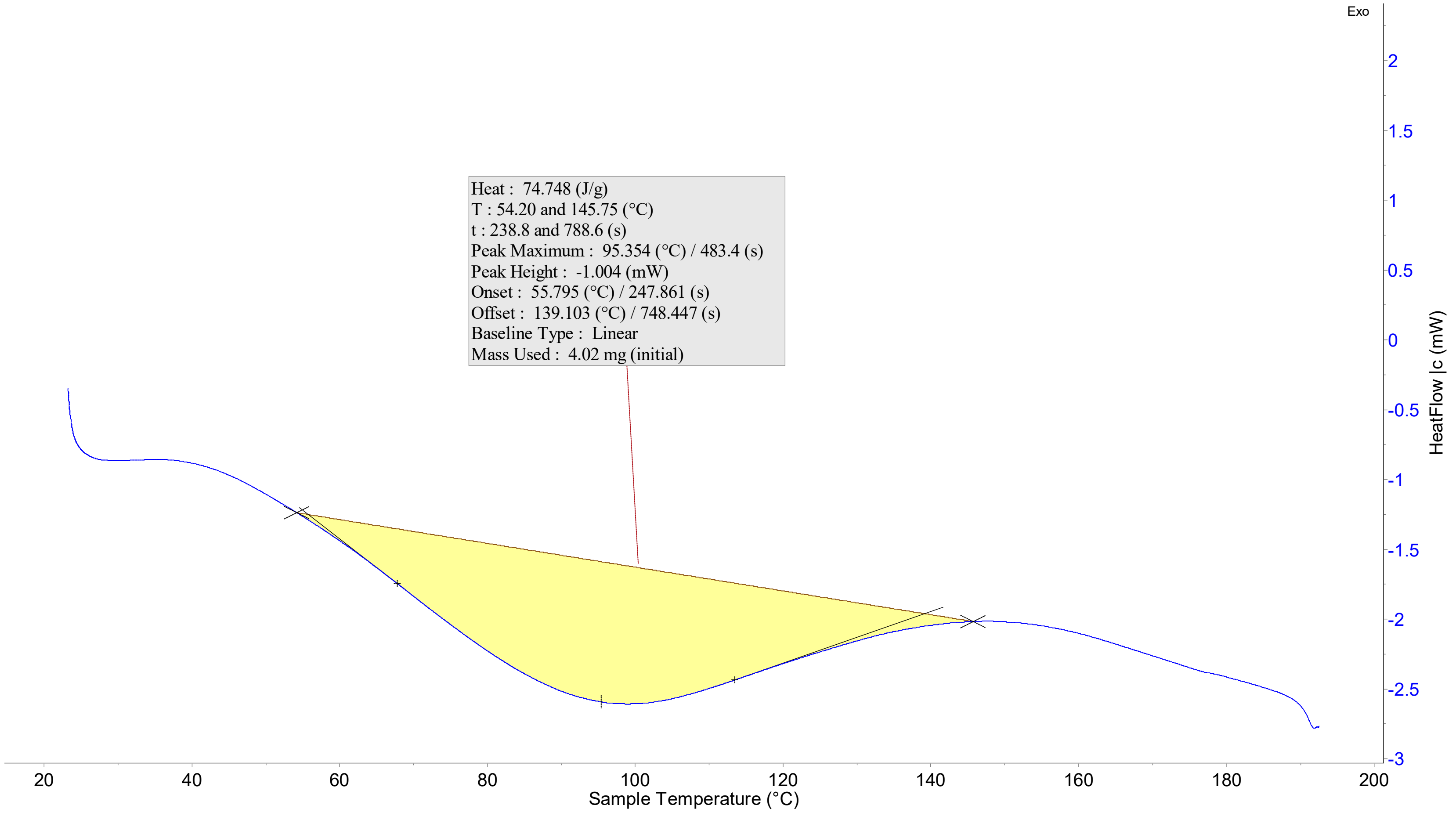

Supplement: Supporting Information — Additional supporting information can be found online in the Supporting Information section. [file 5571705.f1.zip › S7.pdf]
