# Supplementary figures and images for: Formation and Physicochemical Properties of Freeze-Dried Amyloid-Like Fibrils From Pinto Bean Protein: Amyloid-Like Fibrils From Pinto Bean Protein
Source: Int J Anal Chem. 2024 Oct 23;2024:5571705. doi: 10.1155/2024/5571705 (PMC11524705; doi:10.1155/2024/5571705)

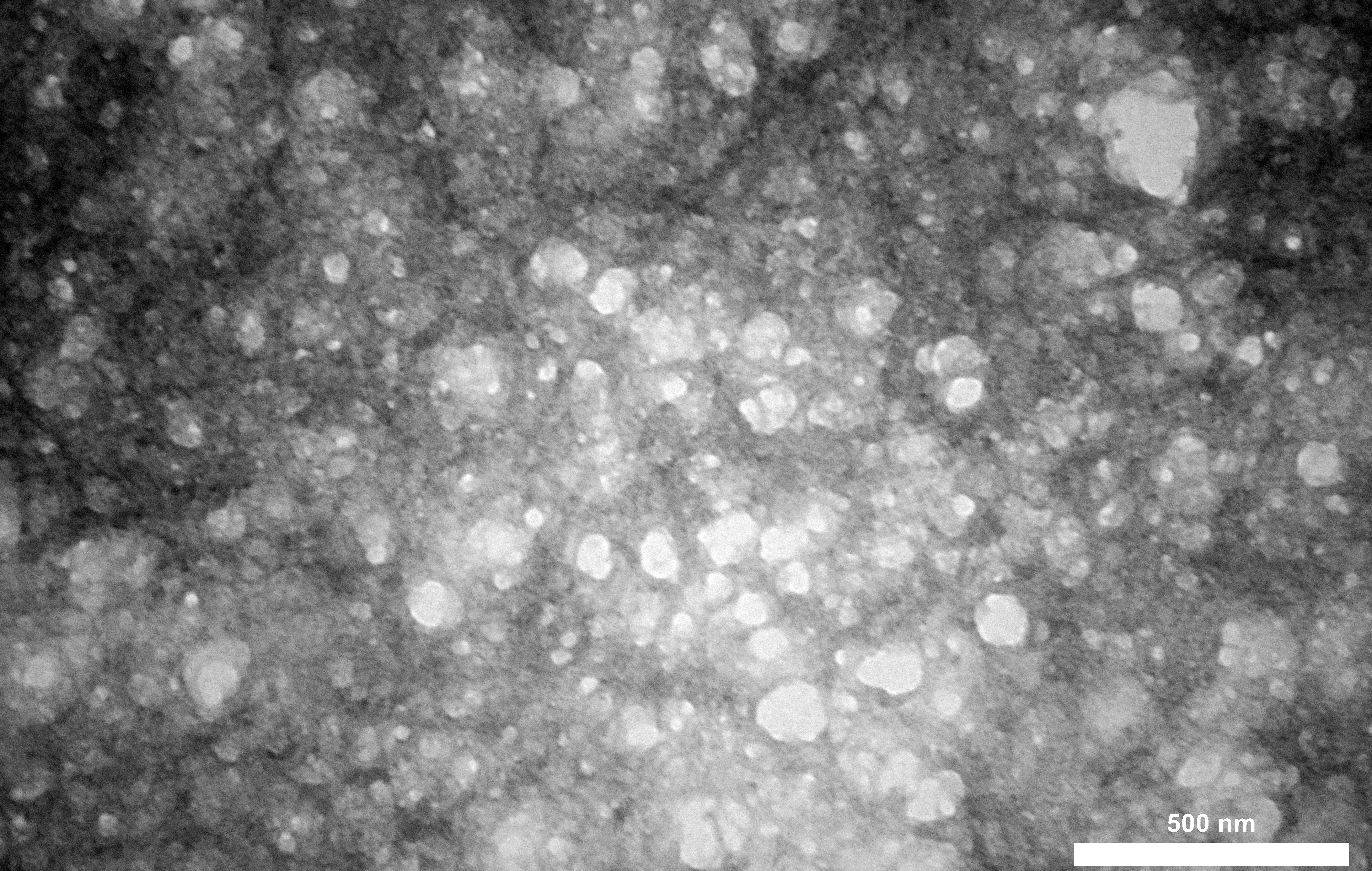

Supplement: Supporting Information — Additional supporting information can be found online in the Supporting Information section. [file 5571705.f1.zip › S1.jpg]

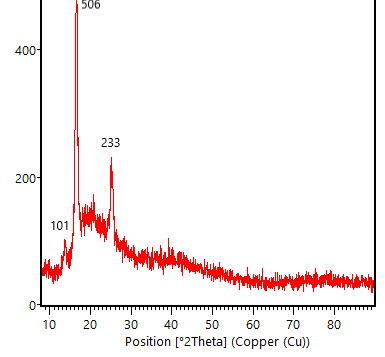

Supplement: Supporting Information — Additional supporting information can be found online in the Supporting Information section. [file 5571705.f1.zip › S10.png]

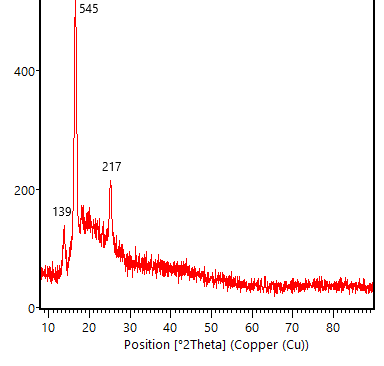

Supplement: Supporting Information — Additional supporting information can be found online in the Supporting Information section. [file 5571705.f1.zip › S11.png]

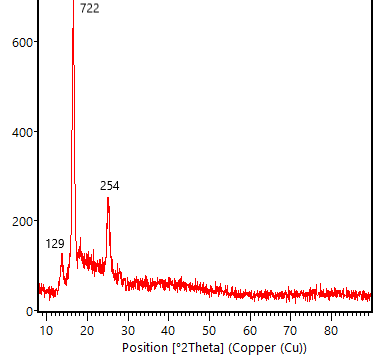

Supplement: Supporting Information — Additional supporting information can be found online in the Supporting Information section. [file 5571705.f1.zip › S12.png]

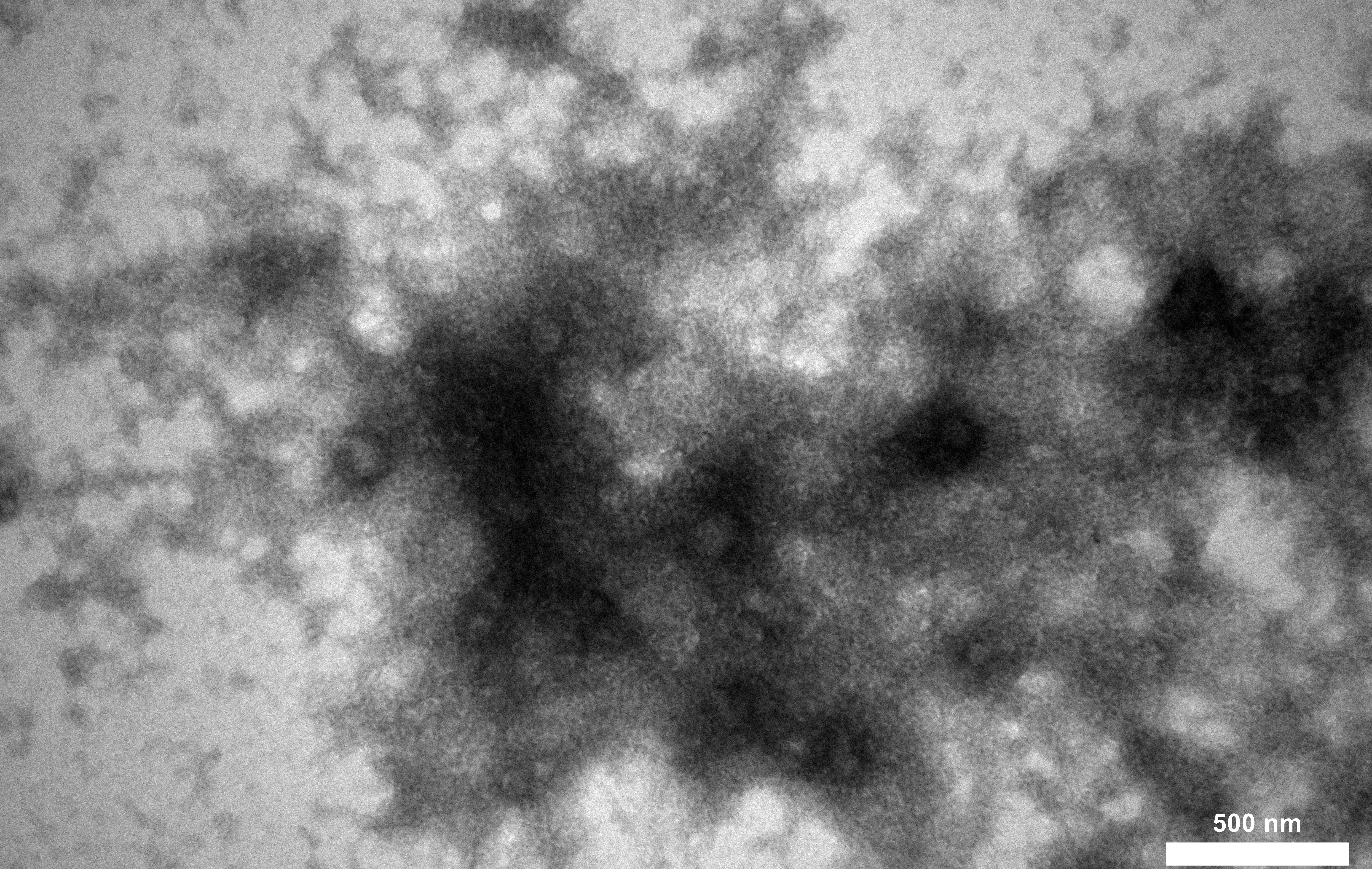

Supplement: Supporting Information — Additional supporting information can be found online in the Supporting Information section. [file 5571705.f1.zip › S2.jpg]

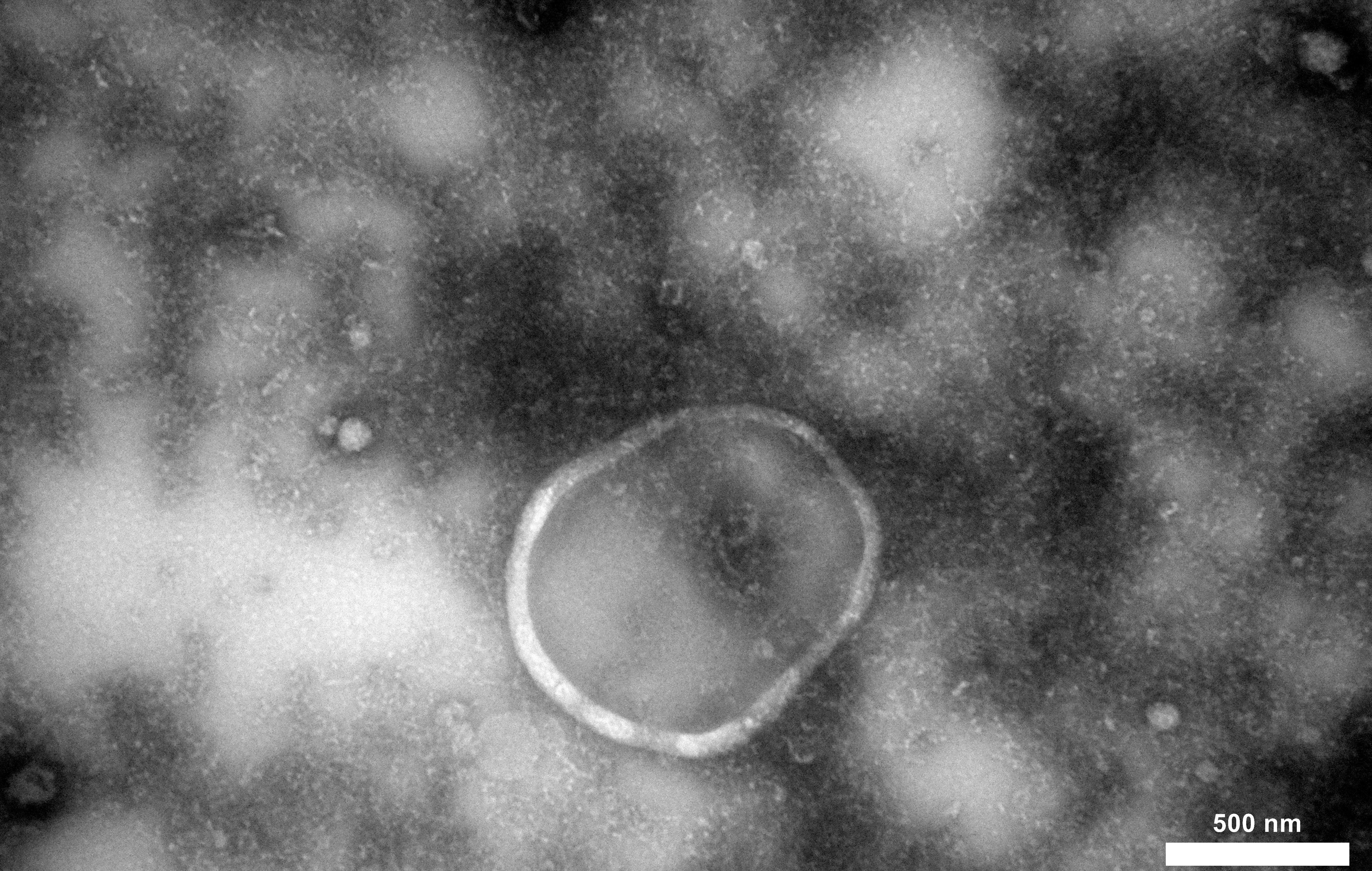

Supplement: Supporting Information — Additional supporting information can be found online in the Supporting Information section. [file 5571705.f1.zip › S3.jpg]

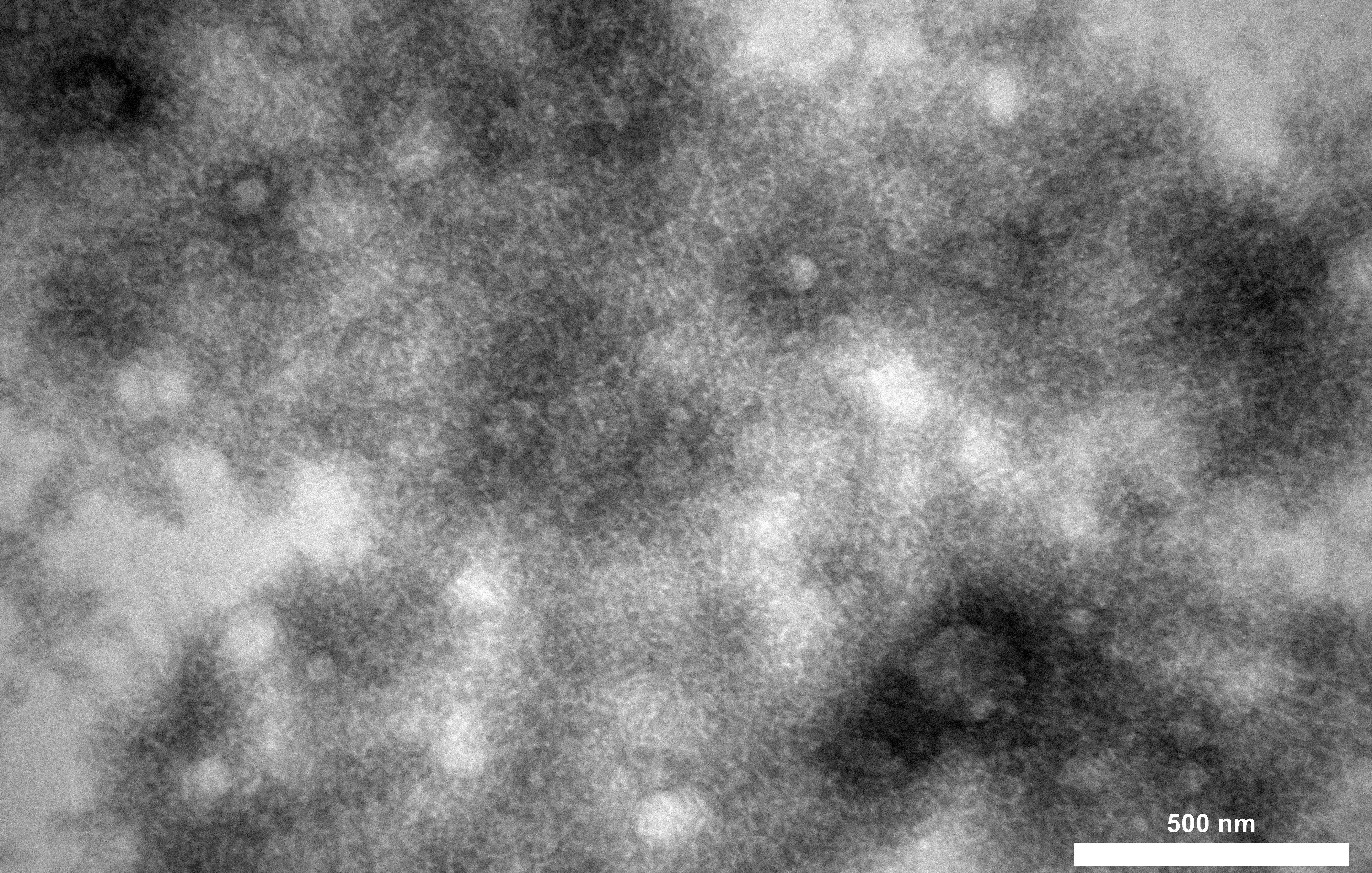

Supplement: Supporting Information — Additional supporting information can be found online in the Supporting Information section. [file 5571705.f1.zip › S4.jpg]

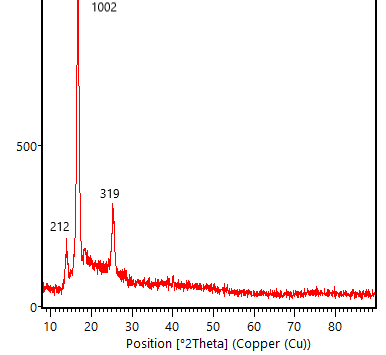

Supplement: Supporting Information — Additional supporting information can be found online in the Supporting Information section. [file 5571705.f1.zip › S9.png]
